# Image Guidance in Radiation Therapy: Techniques and Applications

**DOI:** 10.1155/2014/705604

**Published:** 2014-12-17

**Authors:** Shikha Goyal, Tejinder Kataria

**Affiliations:** Division of Radiation Oncology, Medanta Cancer Institute, Medanta-The Medicity, Gurgaon, Haryana 122001, India

## Abstract

In modern day radiotherapy, the emphasis on reduction on volume exposed to high radiotherapy doses, improving treatment precision as well as reducing radiation-related normal tissue toxicity has increased, and thus there is greater importance given to accurate position verification and correction before delivering radiotherapy. At present, several techniques that accomplish these goals impeccably have been developed, though all of them have their limitations. There is no single method available that eliminates treatment-related uncertainties without considerably adding to the cost. However, delivering “high precision radiotherapy” without periodic image guidance would do more harm than treating large volumes to compensate for setup errors. In the present review, we discuss the concept of image guidance in radiotherapy, the current techniques available, and their expected benefits and pitfalls.

## 1. Introduction

Radiotherapy has always required inputs from imaging for treatment planning as well as execution, when the treatment target is not located on the surface and inspection and visual confirmation are not feasible. Traditional radiotherapy practices incorporate use of anatomic surface landmarks as well as radiologic correlation with two-dimensional imaging in the form of port films or fluoroscopic imaging.

Broadly, imaging has two major roles in radiotherapy:

- Sophisticated imaging techniques such as contrast enhanced computed tomography (CECT) scans, magnetic resonance imaging (MRI), positron emission tomography (PET) scans, and angiography obtain three-dimensional (3D) structural and biologic information which is used to precisely define the target and thus enable precise and accurate treatment planning with shaped beams in isocentric or non-isocentric geometry.
- “In-room” imaging methods (planar, volumetric, video, or ultrasound-based) obtain periodic information on target position and movement (within the same session or between consecutive sessions), compare it with reference imaging, and give feedback to correct the patient setup and optimize target localization. They also have the potential to provide feedback that may help to adapt subsequent treatment sessions according to tumor response.

More specifically, modern day radiotherapy regards the latter application with “in-room” imaging as “image guided radiation therapy” (IGRT).

Modern external beam radiotherapy techniques such as intensity modulated radiation therapy (IMRT), volumetric modulated arc therapy (VMAT), stereotactic radiosurgery (SRS), or stereotactic radiotherapy (SRT) have helped reduce the safety margin around the target volumes thus allowing for lower normal tissue doses without compromising delivery of tumoricidal doses. However, there is a great deal of uncertainty in accurately defining of the position of targets during the delivery of fractionated radiotherapy, both during a given fraction and between successive fractions. Targets that may move during treatment due to respiratory or peristaltic movements or with cardiac pulsations create an even bigger challenge. Hence, there is need to develop and implement strategies to measure, monitor, and correct these uncertainties. This has led to evolution of various in-room imaging technologies which enable evaluation and correction of setup errors, anatomic changes related to weight loss or deformation, or internal organ motion related to respiration, peristalsis, or rectal/bladder filling.

Brachytherapy treatment planning also incorporates orthogonal X-ray imaging and fluoroscopy for guiding brachytherapy catheter/applicator placement, volumetric imaging with CT or MRI for applicator identification and reconstruction, and plan optimization in three dimensions based on imaging. Isodose distribution is reviewed and optimized on visualizing dose distribution to the target as well as critical structures. This adds to treatment efficacy and safety.

## 2. The Concept of IGRT

Though technically complex, surgical procedures enable the operating surgeons to directly visualize and handle their targets, thus eliminating the ambiguity in identification and appropriate management. Radiotherapy, despite being a local therapy that aims to achieve similar goals, inherently carries the disadvantage of making a significant number of assumptions when using traditional treatment techniques. The 3D image dataset acquired at simulation is a snapshot of the tumor, its relation to normal structures, and the patient's shape and position at a single time point, and it is this model that is used for plan development and dose calculation. During the planning stage, a lot of assumptions based on prior experience and literature are used with regard to clinical target volume (CTV) margins to define the microscopic spread around the tumor and planning target volume (PTV) margins to incorporate the expected range of internal organ motion and setup errors. The treatment is then carried out with the belief that all those assumptions would hold true for any given patient during daily treatment sessions, the foremost being that the patient and tumor anatomy and their positions with respect to the positioning devices have remained unchanged since the time of simulation. However, the assumption that the dose calculated on the CT dataset on the planning system matches the dose delivered through each fraction or through the entire radiation therapy course is grossly in error. Additionally, the internal organs and targets move with respiration and peristalsis and planning radiotherapy on a static image dataset is unable to account for errors due to this motion. To ensure that all of these assumptions do not compromise the dose delivery to the CTV, wider PTV margins are taken. This causes a large volume of normal tissues to be included in the irradiated volume. IGRT gives a method to capture this information regularly during the treatment course in the form of serial “snapshots” and is a means of verifying accurate and precise radiation delivery. In simple terms, the IGRT process ensures that the delivered treatment matches the intended treatment in accurately targeting the tumor while minimizing “collateral damage.” Changes to the composite delivered dose and their impact on disease control as well as toxicity may be minimized by use of appropriate localization devices and PTV margins. Occasionally, replanning may be required if gross deviations beyond predetermined tolerances are observed [1–3].

IGRT allows assessment of geometric accuracy of the “patient model” during treatment delivery. It provides a method whereby deviations of anatomy from initial plan are determined and this information is used to update dosimetric assumptions. Correction strategies may include daily repositioning to register patient position in accordance with the base plan or recalculation of treatment delivery in real time to reflect the patient's presentation during a given fraction. This philosophy of reevaluating treatment and accounting for the differences between actual patient anatomy on a given day and the snapshot of planned treatment is known as adaptive radiotherapy [4]. The eventual goal is to reevaluate and in certain situations redefine daily positioning for treatment to keep it on the same track as the intended treatment. Future applications may include dose titration for maximizing effect or mitigating side effects.

## 3. Errors and Margins

An error in radiotherapy delivery is defined as any deviation from intended or planned treatment. A great degree of uncertainty is inherent to radiotherapy practices and may be in the form of mechanical uncertainties related to treatment unit parameters such as couch and gantry motion, patient uncertainties related to ability to lie comfortably in a certain position and cooperate during the treatment time, geometric uncertainties related to position and motion of target, and dosimetric uncertainties. IGRT deals with the geometric uncertainties, which may be either intrafractional or interfractional [5, 6].

Both inter- and intrafractional uncertainties may be a result of a combination of systematic and random errors.

A systematic error is essentially a treatment preparation error and is introduced into the chain during the process of positioning, simulation, or target delineation. This error, if uncorrected, would affect all treatment fractions uniformly. A random error, on the other hand, is a treatment execution error, is unpredictable, and varies with each fraction. Systematic errors shift the entire dose distribution away from the CTV, while random errors blur this distribution around the CTV. Of the two, systematic error is more ominous since it would have a much larger impact on treatment accuracy and hence the therapeutic ratio.

Margins are added to the CTV to take these errors into account. These margins are geometric expansions around the CTV and may be non-uniform in all dimensions depending on the expected errors. These margins ensure that dosimetric planning goals are met despite the variations during and between fractions. ICRU 62 defines the expansion of PTV around the CTV as a composite of two factors—internal target motion (internal margin) and setup variations (setup margin) [7]. Depending on observed systematic and random errors in a given setup for a particular treatment site, a variety of recipes for calculating PTV margins exist in literature [8, 9]. To enhance the therapeutic ratio, a host of correction strategies may be applied to reduce these margins and may include online or offline correction of interfraction errors or real time correction of intrafraction motion. Tracking and correcting organ motion helps reduce internal margin while improved accuracy of positioning reduces setup margins, thus reducing the required PTV margin.

## 4. Offline versus Online Corrections

Offline and online IGRT correction strategies refer to whether the patient is on the treatment couch while the verification is being done and whether the correction would be applied to the same or subsequent sessions.

In the offline strategy, images are acquired before treatment and matched to the reference image at a later time point. This strategy aims to determine the individual systematic setup error and thus reduce it. When combined with setup data of other patients treated under the same protocol, it helps define the population standard error for that treatment in that institution. Widely used offline correction protocols include Shrinking action level and No action level protocols [10, 11]. PTV margins in an institution depend on these determinations of individual and population systematic errors.

An online strategy, on the other hand, employs acquisition of images and their verification and correction prior to the day's treatment. It aims to reduce both random and systematic errors. The treatment site and the expected magnitude of error may determine the frequency of online imaging. Sites where large daily shifts are anticipated (abdomen, pelvis, and thorax) or where even slight shifts will alter the dose distribution within adjacent critical structures (paraspinal tumors, intracranial tumors in close proximity to optic structures) are best managed with daily imaging. Our experience with online corrections showed the maximum errors in thorax followed closely by abdomen and pelvis. The minimum errors were observed in head and neck region [12]. Additionally, treatments such as VMAT and SBRT carry the potential to translate minor shifts into major alterations in dose distribution and hence require daily online verification. For daily online correction, systematic and random errors may be calculated from the matched data. Post-treatment imaging is required to quantify both intrafraction motion and residual errors. If evaluated for a patient population, these data may help check the PTV margin for that treatment protocol. In fact, the use of daily online imaging with corrections in conjunction with use of automatic couch with 6 degrees of freedom has obviated the need for invasive frames for SRS treatments [13].

## 5. IGRT Technology Solutions

Depending on the imaging methods used, the IGRT systems may broadly be divided into radiation based and nonradiation based systems [14, 15].

### 5.1. Nonradiation Based Systems [16–22]

These may employ ultrasound, camera-based systems, electromagnetic tracking, and MRI systems integrated into the treatment room.

#### 5.1.1. Ultrasound-Based Systems

These systems (e.g., BAT, SonArray, Clarity) acquire 3D images that help align targets to correct for interfractional errors. Geometric accuracy is 3–5 mm and the greatest advantage is lack of any ionizing radiation. Sites of common application include prostate, lung, and breast radiotherapy.

#### 5.1.2. Camera-Based (Infrared) or Optical Tracking Systems

These systems identify the patient reference setup point positions in comparison to their location in the planning CT coordinate system, which aids in computing the treatment couch translation to align the treatment isocenter with plan isocenter. Optical tracking may also be used for intrafraction position monitoring for either gating (treatment delivery only at a certain position of target) or repositioning for correction. Tools such as AlignRT image the patient directly and track the skin surface to give real time feedback for necessary corrections. These systems have found application in treatment of prostate and breast cancer and for respiratory gating using external surrogates. Geometric accuracy is 1-2 mm, but application is limited only to situations where external surface may act as a reliable surrogate for internal position or motion.

#### 5.1.3. Electromagnetic Tracking Systems

These systems (e.g., Calypso) make use of electromagnetic transponders (beacons) embedded within the tumor, and motion of these beacons may be tracked in real time using a detector array system. Beacons need to be placed through a minimally invasive procedure, their presence may introduce artifacts in MR images, and there are limitations to the patient size. Calypso has a geometric accuracy of <2 mm, but its use at present is limited to prostate radiotherapy.

#### 5.1.4. MRI-Guided IGRT

These systems (e.g., ViewRay) help real time assessment of internal soft tissue anatomy and motion using continual soft tissue imaging and allow for intrafractional corrections. Geometric accuracy of the system is 1-2 mm. However, MRI has certain drawbacks such as motion artifacts, distortion with non-uniform magnetic fields, and cannot be performed for patients with pacemakers or metallic implants. All these limitations of diagnostic MRI apply to this IGRT system as well. A wide application potential exists in treatment of prostate, liver, and brain, as well as for brachytherapy.

### 5.2. Radiation Based Systems

These include static as well as real time tracking, using either kilovoltage (KV), megavoltage (MV), or hybrid methods.

#### 5.2.1. Electronic Portal Imaging Devices (EPID)

**Figure 1:**
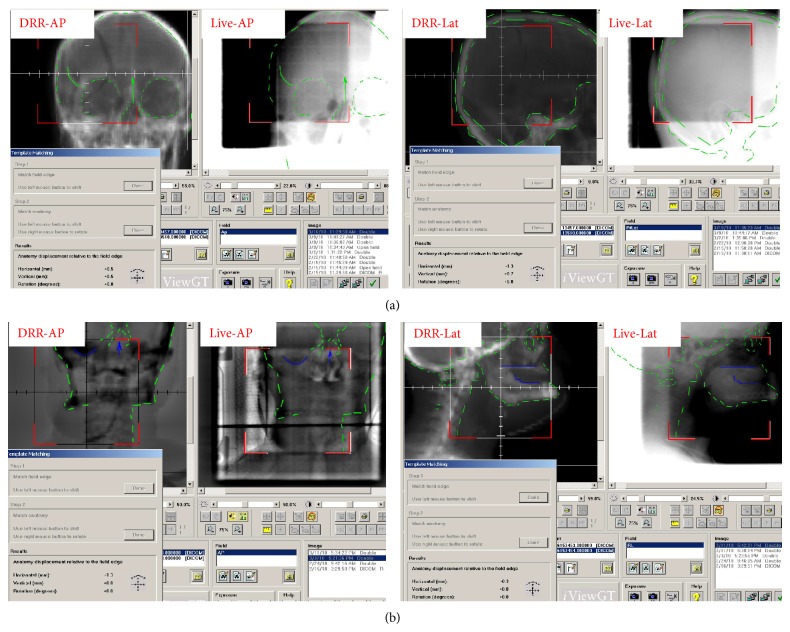
Use of MV EPID for online correction using orthogonal 2D images (anteroposterior and lateral). Both the field and the bony anatomy are matched sequentially to give an estimate of error. The comparison of live image with reference DRR helps assess and correct translational shifts but does not estimate rotational errors. (a) Right parietal glioma. (b) Head and neck cancer. MV: Megavoltage; EPID: Electronic portal imaging device; 2D: two-dimensional; DRR: Digital reconstructed radiograph.

EPID was developed as a replacement of film dosimetry for treatment field verification and is based on indirect detection active matrix flat panel imagers (AMFPIs). They are offered as standard equipment by nearly all linear accelerator (LINAC) vendors as both field verification and quality assurance (QA) tools. Image acquisition is 2D, with a geometric accuracy of 2 mm. Bony landmarks on planar images are used as surrogates for defining positional variations respective to the digital reconstructed radiographs (DRRs) developed from the planning CT dataset (Figure 1). Different systems may use either KV or MV X-rays for imaging, with the image contrast being superior with KV images while there is lesser distortion from metallic implants (dental, hip prostheses) in MV images. EPID systems are unable to detect or quantify rotations. Average dose per image is 1–3 mGy for KV systems while it is as high as 30–70 mGy for MV systems [23–25].

#### 5.2.2. Cone Beam CT (CBCT), KV or MV

**Figure 2:**
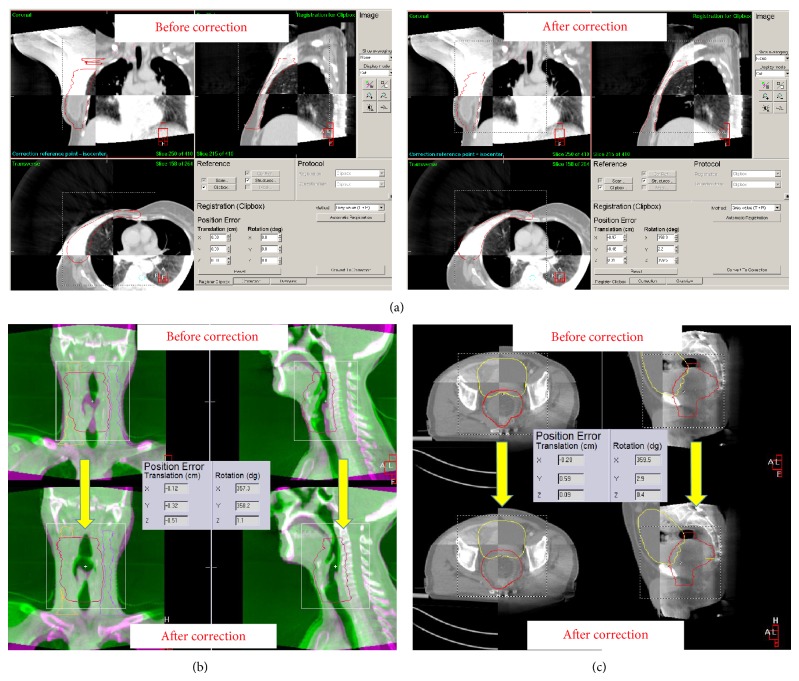
KV CBCT volumetric imaging. Both translational and rotational errors may be estimated. Translational errors are easily corrected whereas few systems have provisions for correcting rotational errors with couch rotations. (a) CBCT compared with reference scan before and after correction of setup error in a case of Carcinoma right breast, post-mastectomy. (b) CBCT correction in a case of Carcinoma larynx. (c) CBCT in a case of Carcinoma prostate not only corrects for setup errors, but also provides an estimate of reproducibility of prostate position with respect to bladder filling. In this particular case, the live image shows negligible bladder filling and treatment was delayed to allow for optimum bladder position for obtaining a reproducible position of prostate as well as moving the bowel out of treatment field. KV: kilovoltage; CBCT: cone beam computed tomography.

These systems consist of retractable X-ray tube and amorphous silicon detectors mounted either orthogonal to (Elekta Synergy, Varian OBI) or along the treatment beam axis (Siemens Artiste). These have capability of 2D, fluoroscopic and CBCT imaging. Another system (Vero, BrainLAB) consists of a gimbaled X-ray treatment head mounted on an O-ring with two KV X-ray tubes, two flat panel detectors, and an EPID. The O-ring can be rotated 360 degrees around the isocenter and can be skewed 60 degrees around its vertical axis. Geometric accuracy is 1 mm or lesser with possibility of 2D and 3D matching with DRRs or X-ray volumetric images generated from planning CT data sets. Scanning is done through a continuous partial or complete gantry rotation around the couch, acquiring the “average” position of organs with respiratory motion. Both interfraction setup changes and anatomical changes related to weight changes or organ filling (bladder, rectum) may be monitored (Figure 2). Repeat scans at the end of treatment may give an estimate of intrafractional changes. For tumors discernible separately from surrounding normal tissue, treatment response may also be monitored and these scans may be used for dose recalculation or treatment plan adaptation after necessary image processing. KV CT gives better contrast resolution compared to MV CT but may be limited by artifacts from prostheses and scatter from bulky patient anatomy. Average dose per image is 30–50 mGy [26–29].

#### 5.2.3. Fan Beam KV CT (CT-on-Rails)

This system has an in-room CT scanner and gantry that moves across the treatment couch/patient, which can be rotated towards either the scanner or the gantry for imaging and treatment, respectively. 3D images are taken with the patient immobilized on the couch, the difference from a diagnostic CT being a larger bore size (>80 cm diameter) to accommodate bulky immobilization devices, and a multislice detector. Accuracy and applications are similar to CBCT with average dose of 10–50 mGy per image [30].

#### 5.2.4. Fan Beam MV CT (TomoTherapy Hi ART II)

This includes an on-board imaging system to obtain MV CT images of the patient in treatment position. The same LINAC is used to generate both the treatment (6 MV) and imaging beam (3.5 MV). A xenon detector located on the gantry opposite the LINAC collects exit data for generation of MV CT images. Patient dose from imaging varies with pitch setting and is typically 10–30 mGy per scan [31].

#### 5.2.5. Hybrid Systems for Real Time 4D Tracking

**Figure 3:**
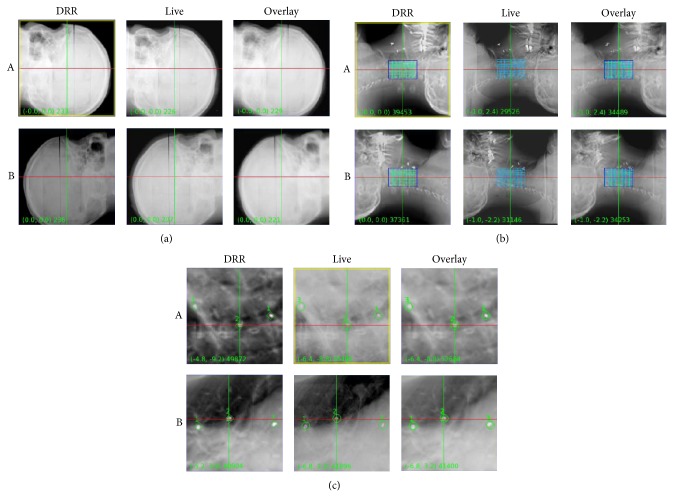
CyberKnife console showing the tumor tracking options in a case of head and neck malignancy. (a) 6D skull for skull base lesions. (b) Spine tracking for paravertebral tumors. (c) Fiducial tracking for all other lesions whose motion is independent of skull or spine position, such as base of tongue or neck nodes.

*2D KV Stereoscopic Imaging (CyberKnife)*. The Accuray CyberKnife robotic radiosurgery system consists of a compact LINAC mounted on an industrial robotic manipulator arm which directs the radiation beams to the desired target based on inputs from two orthogonal X-ray imaging systems mounted on the room ceiling with flat panel floor detectors on either side of couch, integrated to provide image guidance for the treatment process. Images are acquired throughout the treatment duration at periodic intervals ranging from 5 to 90 seconds, and the couch and robotic head movements are guided through an automatic process. Several tracking methods may be used depending upon the treatment site (Figure 3). Skull, skull base, or brain tumors may be treated using 6D skull tracking, paravertebral tumors whose movement parallels that of spine may be treated with X-Sight spine tracking, and lung tumors that are surrounded by normal lung parenchyma may be tracked with X-Sight lung tracking. Lung tracking may employ automatic generation of internal target volume depending upon visibility of tumor through both, one or none of the X-ray imaging systems in the treatment position. For all other tumors (e.g., prostate, liver, neck nodes, abdominal masses, etc.), internal surrogates or fiducial markers may need to be placed within or in direct contact with the tumor and the tumor motion is tracked and corrected for through monitoring the fiducial position including translations, rotations, and deformation. Respiratory motion is also monitored and accounted for when correcting for target position and motion through a synchrony model generated in real time. The system also has a couch that has 6 degrees of freedom to correct for positional variations. Treatment may be limited by patient position and size, and posterior treatment beams cannot be used. A semi-invasive procedure may be required if fiducial markers are needed for tracking. This system can be employed for both cranial (frameless) and extracranial radiosurgery or SRT [32, 33].

*Real Time Tumor-Tracking (RTRT) System*. This system is designed for real time tracking of tumors by imaging implanted fiducials and using this information for gating. It consists of four X-ray camera systems mounted on the floor, a ceiling-mounted image intensifier, and a high-voltage X-ray generator. The LINAC is gated to irradiate the tumor only when the marker is within a given tolerance from its planned coordinates relative to the isocenter [34, 35].

*VERO*. This system has two X-ray tubes and corresponding flat panel detectors and uses a combination of initial couch motion and a pair of radiographs for patient alignment. The couch is capable of 3D alignment for initial coarse setup and then the on-board imaging subsystem helps fine-tuning. A pair of radiographs is acquired and registered with prior DRRs using bony landmarks to evaluate the translational and rotational shifts. The system can also compensate for organ motion [36].

#### 5.2.6. Combination Alignment Systems: Optical Imaging and 2D KV Orthogonal Imaging

*ExacTrac X-Ray 6-D Stereotactic IGRT System*. It uses a combination of optical positioning and KV radiographic imaging for online positioning corrections. There are two main subsystems: an infrared-based system for initial patient setup and precise control of couch movement using a robotic couch and a radiographic KV X-ray imaging system for position verification and readjustment based on internal anatomy or implanted markers. Infrared system may also be used for respiratory monitoring and signaling to LINAC for beam tracking and gating. Novalis Tx combines this system with an additional on-board imaging system (MV, KV X-rays, and KV CBCT) on a multiphoton/electron beam LINAC [37, 38].

## 6. Guidelines for Medical Personnel and Implementation

American College of Radiology (ACR) and the American Society for Radiation Oncology (ASTRO) jointly developed guidelines for IGRT that define the qualifications and responsibilities of personnel including radiation oncologists, medical physicists, dosimetrists and radiation therapists, QA standards, clinical implementation, and suggested documentation. Similar guidelines have also been proposed by European agencies [39–41]. A summary of the key points is given below.

### 6.1. Qualifications and Responsibilities

*Qualifications*. Respective personnel should obtain appropriate certification with specific training in IGRT before performing any stereotactic procedures.

*Responsibilities*

*Radiation Oncologist*. (i) Conduct of disease-specific treatment, staging, evaluation of comorbid conditions and prior treatments, exploration of all available treatments including discussion of pros and cons of IGRT, treatment, and subsequent follow-up.

(ii) Determination of the most appropriate patient positioning method, recommendation of the appropriate approach to manage organ motion, supervision of simulation paying particular attention to positioning, immobilization and appropriate motion management, determination and delineation of target volumes and relevant normal critical structures using available imaging techniques, communication of expected goals and constraints and collaboration with the physicist in the iterative process of plan development to achieve the desired goals, supervision of treatment delivery and determination of acceptable day-to-day setup variations, and participation in the QA process and subsequent approval.

*Medical Physicist*. (i) Acceptance testing and commissioning, assuring mechanical, software, and geometric precision and accuracy, as well as image quality verification and documentation in a given IGRT system.

(ii) Implementation and management of a QA program.

(iii) Development and implementation of standard operating procedures (SOPs) for IGRT use, in collaboration with the radiation oncologist.

*Dosimetrist*. (i) Normal structure delineation under the guidance of radiation oncologist.

(ii) Management of volumetric patient image data (CT and other fused data sets) on radiation treatment planning (RTP) system.

(iii) Generation of a treatment plan under oncologist's and physicist's guidance.

(iv) Generation of all technical documentation for IGRT plan implementation.

(v) Assisting with treatment verification.

*Radiation Therapist*. (i) Understanding and appropriate use of immobilization/repositioning systems.

(ii) Performance of simulation and generation of imaging data for planning, implementation of treatment plan, acquisition of periodic verification images under supervision and periodic evaluation of stability and reproducibility of the immobilization/repositioning system, and reporting inconsistencies immediately.

### 6.2. IGRT Implementation

*Fiducial Markers*. These serve as surrogates to soft tissue targets when they are difficult to visualize and their alignment cannot be related to bony anatomy. These may be tracked in real time to obtain 3D coordinates of the target for subsequent corrections.

*Moving Targets and Delineation*. Intrafraction target motion or interfraction displacement, deformation, or alteration of targets and other tissues should be accounted for during determination of PTVs. Appropriate motion management methods should be chosen depending on available expertise and degree and type of motion. This process starts at the time of simulation and continues throughout till the end of therapy.

*Patient Positioning*. It is imperative to ensure the accuracy of patient position and its reproducibility for fractionated treatments relative to the chosen IGRT device as well as treatment unit.

*Image Acquisition*. The IGRT system should be calibrated to ensure high imaging quality with attention to slice thickness uniformity, image contrast, spatial resolution, isocenter alignment between imaging and treatment planning and delivery systems, accuracy of software used for identification, and correction of couch misalignments. Relevant QA procedures should ensure reliability and reproducibility of the entire process.

*Treatment Verification*. Image review by radiation oncologist at the first fraction and then periodically is necessary to ensure treatment accuracy and reproducibility. Each department should determine its own threshold of couch positioning changes that would necessitate setup review or change before treatment delivery.

*Quality Assurance and Documentation*. A documentation of all the necessary QA procedures throughout the course of simulation, treatment, and periodic verification should be maintained. These would help determine departmental thresholds for action as well as serve as guides for modification of the processes involved following review of findings.

## 7. IGRT: Clinical Benefits

Use of the IGRT process has improved our awareness and understanding of daily inter- and intrafractional setup variations and motion. Real time tracking has helped quantify interpatient and intrapatient variations in lung and liver tumor motion related to breathing and complexities of such motion have become clearer. We now understand that even when breath-holds are repeated, the relative position of soft tissue and skeletal structures may vary, rendering use of bony landmarks useless for such endeavors. Changes in prostate position (translation, rotation, and shape) have been quantified and we can better correct for these errors as well as tailor PTV margins to these findings, thus allowing more accurate targeting. Understanding of the various IGRT techniques, their applicability, limitations, and additional radiation hazards helps the radiation oncologist take an educated decision on the method best suited to a particular clinical situation for maximizing benefit from radiation therapy. Changes in parotid position relative to the tumor in head and neck cases, change in body contour due to weight loss, seroma, or body fluid collections, change in prostate position relative to bladder or rectal filling and effect of bowel gas, reduction of tumor size during treatment, and changes in spinal position during spinal or head and neck radiotherapy are situations which were never even considered of significance in the pre-IGRT era and their respective roles and solutions are being developed as we are understanding their role during treatment. With better geometric precision, volume of irradiated healthy normal tissue can be significantly reduced with reduction in toxicity risks. Adaptation to reduction in tumor volume may lead to additional gains in normal tissue toxicity reduction.

Results from ongoing and future trials will hopefully demonstrate the net gain in therapeutic ratio from application of IGRT technologies and the onus lies on the radiation oncology community to take up the challenge of demonstrating the benefit of these potentially expensive approaches.

IGRT is most likely to benefit clinical situations where the tumor is in close proximity to sensitive healthy tissues, when doses required for disease control exceed the tolerance levels of adjacent normal tissues or when large organ motion and setup errors may result in severe consequences of positional errors. All patients treated with conformal radiotherapy, IMRT, and SBRT should, in theory, benefit from IGRT. Thoracic and upper abdominal targets with significant respiratory motion, obese patients, head and neck cancers, paraspinal and retroperitoneal sarcomas, and prostate cancer are situations that are expected to derive maximum benefit with some clinical experience forthcoming. Clinical situations where even low dose irradiation produces excellent local control, palliative radiotherapy delivered using large fields, and superficial tumors that are amenable to direct visual inspection are likely to derive least benefit from IGRT.

## 8. Concerns with IGRT

Limited availability of experienced trained staff is a major hurdle in wide application of the technique despite its demonstrable benefits, even with the simplest approaches. Other factors that need consideration include quality control, algorithms that define the decisions whether to change a plan or continue with original plan, and need for commercial development of software as well as hardware to match clinical needs and demands. Another major concern regarding frequent on-treatment imaging is the radiation dose to normal tissues. Although the doses from IGRT appear insignificant, only long term follow-up will define any potential risk of second malignancies from low dose exposure. Thus, there is an ongoing debate on the necessary frequency of verification imaging especially when using ionizing radiation. Recent developments in MR-LINACs have tried to address these concerns while allowing daily imaging for treatment verification. Another concern is that of treatment safety since the technologies available in the clinic require integration of hardware and software from different vendors. Clinical use of any system should be preceded by proper acceptance testing, commissioning, and routine QA used to assure accurate regular functionality. Education of all users (oncologists, physicists, and technologists) on safe use and clinical utility is mandatory, along with knowledge of additional dose and possible risks associated with use. No single technology is ideal in every scenario and no single institution can manage to integrate all or most technologies in one place. Only time will tell which of these methods gain wider popularity and acceptance, based on clinical relevance and ease of use.

## 9. Clinical Applications: Current and Future

Use of IGRT systems is essential to treatment of any site where setup deviations and organ motion are anticipated. Additional gains are monitoring of treatment response, weight changes, and organ filling on day-to-day basis. With improved precision of planning systems, use of SRS or SRT, and high dose hypofractionated regimens, the chances of small deviations leading to significant errors in treatment delivery are much higher, and the use of IGRT is far more critical in these situations. Integration of LINACs with MR-based soft tissue imaging and PET-based biological imaging may help even further improve targeting accuracy in the future [42, 43]. However, it is mandatory to ensure proper training of staff and QA at all steps for optimum use of such technology and its integration into routine use.
